# Contextual Specificity in Peptide-Mediated Protein Interactions

**DOI:** 10.1371/journal.pone.0002524

**Published:** 2008-07-02

**Authors:** Amelie Stein, Patrick Aloy

**Affiliations:** 1 Institute for Research in Biomedicine (IRB), Barcelona Supercomputing Center (BSC), Barcelona, Spain; 2 Institució Catalana de Recerca i Estudis Avançats (ICREA), Barcelona, Spain; Yale University, United States of America

## Abstract

Most biological processes are regulated through complex networks of transient protein interactions where a globular domain in one protein recognizes a linear peptide from another, creating a relatively small contact interface. Although sufficient to ensure binding, these linear motifs alone are usually too short to achieve the high specificity observed, and additional contacts are often encoded in the residues surrounding the motif (i.e. the context). Here, we systematically identified all instances of peptide-mediated protein interactions of known three-dimensional structure and used them to investigate the individual contribution of motif and context to the global binding energy. We found that, on average, the context is responsible for roughly 20% of the binding and plays a crucial role in determining interaction specificity, by either improving the affinity with the native partner or impeding non-native interactions. We also studied and quantified the topological and energetic variability of interaction interfaces, finding a much higher heterogeneity in the context residues than in the consensus binding motifs. Our analysis partially reveals the molecular mechanisms responsible for the dynamic nature of peptide-mediated interactions, and suggests a global evolutionary mechanism to maximise the binding specificity. Finally, we investigated the viability of non-native interactions and highlight cases of potential cross-reaction that might compensate for individual protein failure and establish backup circuits to increase the robustness of cell networks.

## Introduction

Proteins are key players in virtually all biological events that take place within and between cells. And yet, proteins seldom act in isolation and often accomplish their function as part of large molecular machines, whose action is co-ordinated through intricate regulatory networks of transient protein-protein interactions. Consequently, much effort has been devoted to unveiling protein interrelationships in a high-throughput manner, and recent years have witnessed the consecution of the first interactome drafts for several model organisms, including human [1], [2].

However, high-throughput interaction discovery experiments indicate only that two proteins interact, but do not provide information about the molecular details or the mechanism of the interaction. Currently, this atomic level of detail can come only from high resolution three-dimensional (3D) structures, where the residue-contacts are resolved and the protein interaction interfaces characterised [3]. By exploring all interactions of known 3D structure as stored in the Protein Data Bank (PDB) [4] we could divide protein interactions into two main categories on the basis of their contact interfaces: domain-domain and domain-peptide interactions [3]. Domain-domain interactions involve the binding of two globular domains from different proteins, thereby creating a large contact interface of 2.000Å^2^ [5] on average. In domain-peptide interactions a globular domain in one protein recognises a short linear motif from another protein, creating a relatively small interface. Such interactions are found predominantly in signalling and regulatory networks [6] and, due to their transient nature, are much more difficult to handle biochemically.

Linear motifs are short patterns of around 10 residues with a common function (i.e. binding to a globular domain) that occur in otherwise unrelated proteins. In isolation these motifs bind their target proteins with sufficient strength to establish a functional interaction. They are frequently found in disordered or unstructured regions, which are now known to be not simply loops or linkers, but serve a variety of functions [7], [8], and adopt a well-defined structure only upon binding. Usually just a few residues in the motif are fixed to a specific amino acid, or restricted to a small set of residues while several positions may be arbitrary (represented either by an ‘*x*’ or a ’.’). For example, Src-homology-3 (SH3) domains bind proline-rich peptides, and several variants of the PxxP pattern have been observed, like [RKY]xxPxxP (class I; square brackets denote several possibilities for a position) and PxxPx[KR] (class II) [9].

Recently, large-scale experiments for the determination of peptide recognition profiles of interaction domains, and derivation of the corresponding patterns, have been developed [10], [11]. Nevertheless, transient peptide-mediated interactions are still underrepresented in high-throughput experiments [12]. Most of what is currently known about this type of interactions is compiled in the Eukaryotic Linear Motif Database (ELM) [13], which provides a literature-curated collection of motifs and their interaction partners. The motifs in ELM usually have between 4 and 11 residues.

Although the binding is mediated by a small number of contacts formed by the residues in linear motifs, this type of interaction is extremely specific *in vivo*. For instance, Lim and co-workers showed that the Pbs2 peptide is recognised only by the SH3 domain of Sho1 (its biological partner) and does not cross-react with any of the other 26 SH3 domains in yeast [14], although interactions with SH3 domains from other species are biophysically possible. More recently, Stiffler *et al.* have also shown that the binding specificity of PDZ domains is optimised across the 157 domains contained in the mouse proteome [15]. However, bonds between residues in linear motifs and globular domains, while sufficient to ensure binding, are too few to explain the high degree of specificity observed *in vivo*. It is thus, as happens in phosphorylation events [16], the biological *context* which ultimately determines interaction specificity.

**Figure 1:**
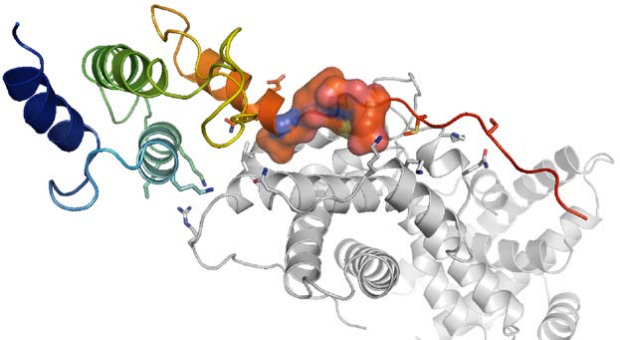
Example of contextual specificity. Interaction between the human retinoblastoma protein (grey) and the Simian virus 40 large T antigen (rainbow) (PDB id 1gh6 [18]). The consensus binding motif [LI].C.[DE] is shown in surface representation, and context residues as sticks.

This context has several aspects –subcellular localization or expression patterns will determine whether proteins that are potential competitors for an interaction *in vitro* actually meet *in vivo* and thus evolve into niches of molecular recognition that allow them to bind only the desired target domain. For instance, in T-cells, the SH3 domain of Fyn does not compete with the GYF domain of CD2BP2 for the proline-rich motif in the cytoplasmic tail of CD2, because Fyn is located in the lipid rafts while CD2BP2 occurs in the detergent-soluble membrane fraction, although the interaction would be possible in *in vitro* assays [17]. Nevertheless, even within a cellular compartment, several interaction domains and their complementary ligands are regularly expressed at the same time, so more contextual information is required to achieve the specificity observed. This information is, to a great extent, contained in the residues surrounding the motif. From here on, *context* refers to those residues in the protein containing the linear peptide that interact with a globular domain in another protein but lie outside the motif, as defined in ELM (Fig. 1).

Many examples in the literature highlight the relevance of the context. For instance, mutations in the LxCxE motif of the SV40 large T antigen binding to the human retinoblastoma protein abolish complex formation, while mutations in the context, even in regions sequentially distant from the motif, still allow binding but diminish or abolish the function [18] (Figure 1). Studies on interactions between the enabled/vasodilator-stimulated phosphoprotein homology 1 (EVH1) and its binding polyproline motif have shown that residues flanking the motif are also crucial in determination of specificity [19]. Other examples include nuclear receptors and co-activator peptides, for which residues adjacent to the defined LxxLL motif, and those in the globular domain outside the motif binding groove, modulate binding affinity and specificity [20].

Here we systematically identify all instances of peptide-mediated protein interactions of known 3D structure, based on the motif patterns collected in ELM, and use them to explore the individual contribution of motif and context to the global binding energy. We also examine a potential global evolutionary mechanism to increase the binding specificity in this type of interaction, and highlight potential cases of cross-talk involving non-native interaction protein pairs.

## Results

### Peptide-mediated interactions of known structure

**Figure 2:**
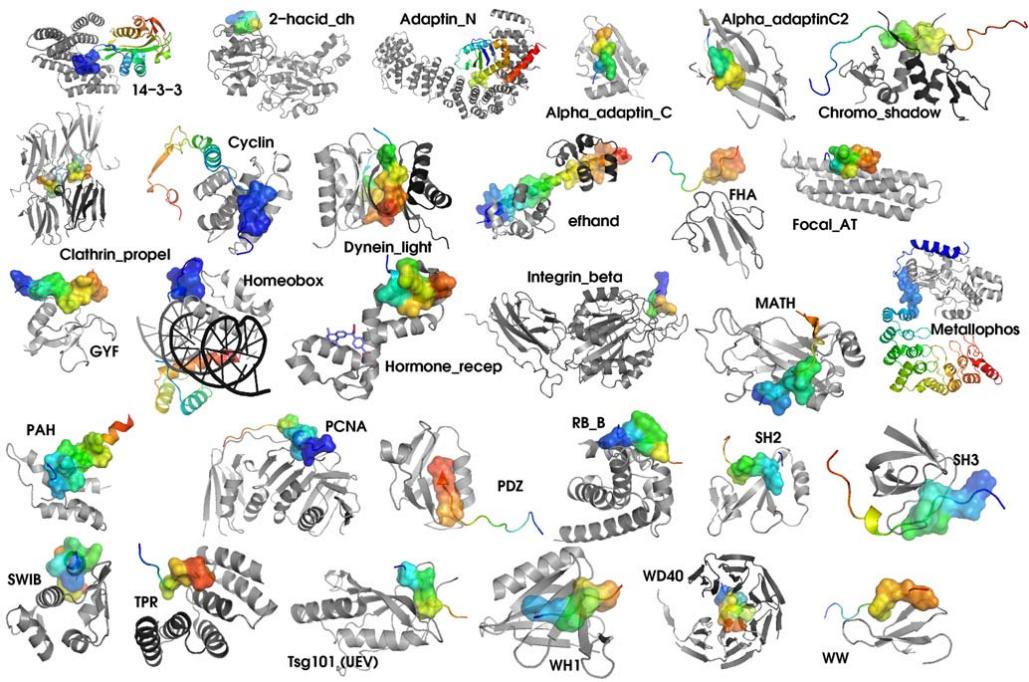
Representative structures for the different types of peptide-mediated protein interactions. Globular domains as defined in Pfam are shown in grey and binding proteins in rainbow colours. Consensus binding motifs are always shown in surface representation.

We extracted information on the 66 consensus motifs responsible for mediating protein interactions, as stored in ELM, to search the over 45,000 structures in the PDB. Our initial automated procedure identified a total of 13,000 potential matches of the annotated motifs, 2,200 of which fulfilled our geometrical criteria (see Materials and Methods) to become peptide-domain interaction candidates. After manual filtering for the correct interaction topology, we identified high-resolution 3D structures of 810 ELM motifs interacting with their binding domains in 611 protein pairs, which represent 47 motifs and 30 globular domains annotated in the ELM database (Figure 2). We then clustered all interacting pairs on 100% sequence identity of both proteins and ended up with a set of 383 non-redundant interactions of known 3D structure.

During visual inspection of all potential domain-peptide interactions we identified 7 distinct motifs in interactions of known structure that did not match any of the patterns collected in ELM so far, binding to the domains 14-3-3, MATH, PDZ (3 new motifs) and SH2 (2 new motifs). Like the known motifs, they bound the appropriate pocket in the domain and contained amino acids similar to key residues in the described patterns. We thus included them in our analysis, thereby extending our set to 390 interactions of known structure (Table S1).

### Contribution of motif and context to binding energy

We found that, as expected, the strongest contribution to the interaction came from contacts involving residues in the motif, responsible, on average, for 79% of the global binding energy, ranging from 12% to 99.7% in the different types of interaction (Table 1). Nevertheless, the contacts outside the motifs were also significant, with an average contribution of the context of 21%. However, surprisingly, our *in silico* alanine scanning analysis showed that truncation of side chains in the motif almost always impaired binding, while a truncation of side chains or residues replacements in the context improved the overall interaction energy in about 20% of our cases. Motifs found within globular domains, such as those binding Metallophos/PP1 or Adaptin_N, showed an extremely high contextual contribution. Very low contextual contributions were observed only in structures with little context; considering only cases with a reasonable amount of context – at least as much as the motif – indicated that the contextual contribution to binding was at least 5%. It is important to note that, to avoid flexibility problems during crystallisation, some of the 3D structures of domain-motif interactions were solved with only a fragment of the motif-containing interaction partner or synthetic peptides to study binding properties, and thus the contribution of the context may be artificially limited. Individual contributions for each family are given in Table 1.

**Table 1:** Motif and context binding contribution for each type of interaction.

domain name	number of cases	number of clusters	% average context contribution	average context length
14-3-3	16	9	20.4±33.5	6.0±10.4
2-Hacid_dh	1	1	28.8±0.0	1.0±0.0
Adaptin_N	6	1	88.1±2.2	57.2±0.9
Alpha_adaptinC2	1	1	4.0±0.0	1.0±0.0
Alpha_adaptin_C	5	4	8.7±5.1	1.8±1.2
Chromo_shadow	2	1	34.3±22.6	8.5±0.5
Clathrin_propel	20	11	16.7±25.3	1.0±0.9
Cyclin_N	21	13	19.2±17.7	2.6±3.8
Dynein_light	2	1	29.0±28.4	4.0±0.0
EFhand	232	67	24.5±16.6	9.4±5.8
FHA	9	4	44.0±26.6	3.8±0.4
Focal_AT	7	3	5.9±4.2	2.4±0.7
GYF	1	1	0.6±0.0	0.0±0.0
Homeobox	2	2	4.4±3.5	2.5±0.5
Hormone_recep	152	72	9.3±7.9	3.0±1.6
Integrin_beta	1	1	33.5±0.0	1.0±0.0
MATH	37	16	16.9±15.3	2.1±1.9
Metallophos	1	1	71.0±0.0	53.0±0.0
PAH	4	3	12.9±5.3	4.0±1.4
PCNA_C	9	9	28.3±18.0	10.1±8.1
PCNA_N	9	8	28.3±18.0	10.1±8.1
PDZ	38	31	25.1±23.6	4.7±6.9
RB_B	2	2	27.1±8.4	8.0±5.0
SH2	77	49	19.0±16.8	3.0±3.0
SH3_1	95	40	29.6±19.3	3.4±3.1
SWIB	3	3	7.4±5.7	3.0±1.6
TPR_1	28	14	25.4±16.6	2.7±1.7
TPR_2	1	1	20.0±0.0	1.0±0.0
Tsg101	2	1	56.5±1.5	4.5±0.5
WD40	12	6	9.0±0.7	1.0±0.0
WH1	4	4	0.3±0.4	0.3±0.4
WW	10	10	19.5±20.5	3.3±2.7
**Total**	**810**	**390**	**21.1**±**19.4**	**5.5**±**7.1**

Overview of the binding contribution per family: Pfam name of the globular domain, number of interacting structures and non-redundant structures identified in the PDB, % binding energy contributed by the context, and the average length of the context, in number of residues.

We also identified several cases of unusual contextual contribution. For instance, the current motif definition for binding to the forkhead-associated domain (FHA) is 4 residues long and starts with pT, which is known to be crucial for the interaction. However, our data shows that 2–4 residues N-terminal of the pT also contributed strongly to the interaction. Experiments by Durocher *et al.* have shown that residues both N- and C-terminal of the pT are crucial for specificity [21]. Taken together, these results suggest that some N-terminal residues should be included in the consensus motif. Indeed, the ELM description of the FHA-binding motif has been updated in the meantime and now also includes two residues N-terminal to pT. It might be, however, that this extension still is not sufficient: a study by Byeon *et al.* showed that while the pT is crucial, the surrounding region of 44 residues is required for tight binding to the FHA domain of Ki67, while short peptides were not sufficient to establish an interaction [22]. Besides conferring a higher specificity, in this case, the context has also been shown to play an important role in the regulation of the protein and its interactions, with three phosphorylation sites identified in it [22].. Note that this structure is not included in our result set because it matches neither the previous nor the current FHA-ligand pattern given in ELM. The need for longer peptides in determining specificity was also raised by Mahajan *et al.*, who studied the interaction between FHA in Rad53 and a 10-residue peptide from Mdt1 in detail, and pointed out that peptide library screens can only provide leads for specificity of (signalling) domains because ionic interactions appear more important in such small peptides then they might be in full-size proteins [23]. Similarly, for Tsg101 we found 9 residues that contributed equally to the binding, although only 4 of them are described to form the motif. Studies on other instances of this interaction are required in order to determine whether these residues are crucial to establish binding and should hence be part of the motif. We also found unexpected results in the interaction between the regulatory subunit MYPT1 and protein phosphatase 1 (PP1), where it has been experimentally shown that the contribution of the motif in formation of the PP1:MYPt1 complex is fundamental [24]. Yet our calculations showed an exceptionally low contribution of about 30% of the motif to the total binding energy. The motif may play a crucial role in the binding process but, due to the very large interface between PP1 and MYPT1, it is difficult to properly infer its importance from the final crystal structure.

The interaction between the EF hand domain and the IQ ligand, while also described as a domain-motif interaction, differed in several ways from other domain-motif interactions studied here: it involved the simultaneous binding of at least two domains, which have different orientations towards the peptide. In additionally, with up to 20 residues the motif is unusually long, while the EFhand domain has only about 30 residues. This atypical combination of size and stoichiometry may explain why the behaviour of this interaction type differs. It is also possible that the combination of several domains helps to achieve high specificity.

To analyse whether the method of structure determination introduces any bias in context contribution, we separated our curated set of domain-motif interactions by methods and computed the contextual contribution to binding for each of them. The vast majority of interactions (over 500) were determined by X-ray crystallography, about 100 by NMR and 150 by other methods. However, we did not observe significant differences between these sets.

We sought for a relationship between contribution to the global binding energy of each residue and their level of sequence conservation within their respective domain families. Among the globular domains involved in peptide-mediated interactions, we found no correlation between sequence variability and energy contribution. We observed a weak trend indicating that domain residues contacting mostly residues in the linear motif were more conserved than those contacting the context. This effect was present in the sequences derived from our dataset, but disappeared when we extended the alignment to other homologous sequences. Therefore it is difficult to unequivocally determine whether this increase in conservation is a true feature of motif-binding residues. The situation differed however when we considered residues in the linear motif. Here we found that the contribution of motif positions with fixed residues to the binding energy was higher than that of restricted positions, which in turn was higher that that of arbitrary positions, with each residue contributing 25%, 21% and 13% of the total energy, respectively (Figure S1). However, the caveat of the large standard deviations observed for all three groups (±17–24%) must be considered.

### Binding vs specificity, from topological and energetic perspectives

The results above show that interaction contacts involving residues in the motifs were nearly optimal, with respect to maximising binding affinity, whereas changes in the context can improve it. This observation suggests that the context has not been selected to increase the strength of the binding, but to prevent non-native peptide-mediated interactions within an organism, to maximise binding specificity. To further test this hypothesis, we studied the topological and energetic compatibility of linear motifs and context residues in the peptide-mediated protein interaction pairs identified here.

To analyse the topological binding variation within each interaction type in more detail, we optimally superimposed the equivalent globular domains (e.g. SH3, PDZ, etc) and used the obtained rotation and translation matrix to calculate the root mean square deviation (RMSD) between motif and context residues in the partner proteins. The placement and orientation of all motifs interacting with a given family was very similar, with an average RMSD of 2.5±3.2Å. The situation differed when we considered the context: We found several distinct topologies for each type of interaction, with contextual contacts being widely spread around the motif-binding groove, resulting in a larger average RMSD of 4.2±4.4Å between the equivalent contacting residues in the partner proteins (Figure S2). Since motif and context RMSD distributions did not show a normal Gaussian behaviour, standard deviations could not be used to compare them properly. We thus applied the Kolmogorov-Smirnov test, which confirmed that these two distributions were significantly different, with a p-value <2.2·10^−16^. Please, note that being closely related proteins, all members of each globular domain families in this work present very similar structural scaffolds, as defined in the Structural Classification Of Proteins (SCOP) database [25]. The interaction topology between a domain and the binding motif was also roughly conserved, with all motifs placed in the same binding groove.

**Figure 3:**
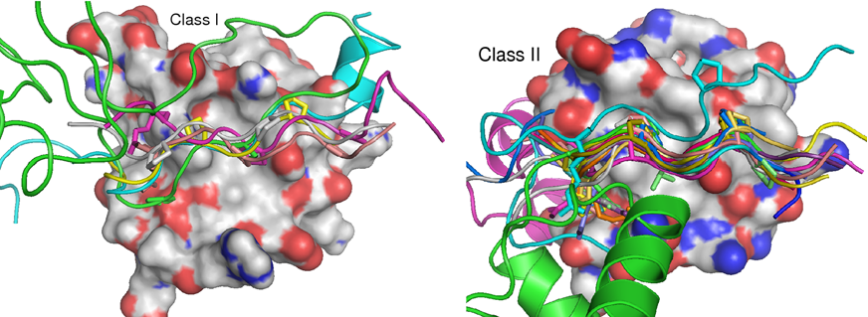
Topological variation of peptides binding human SH3 domains. One domain is shown for each of the two possible orientations of SH3-binding peptides, in surface representation. Native and non-native peptides for class I (pattern [RKY]xxPxxP, left) and class II (pattern PxxPx[KR], right) are shown as ribbons. Key residues are highlighted as sticks. The first and last highlighted residues delimit the motifs; everything N- and C-terminal of that, respectively, is context. Both domains have a similar orientation in the figure, so, as the peptides have opposite orientations, the N-termini of the class I peptides are on the left, while those of the class II peptides are on the right.

**Figure 4:**
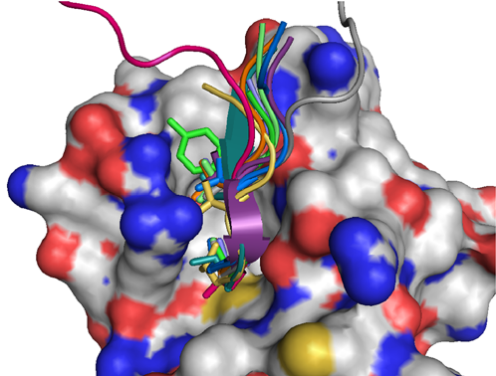
Topological variation of peptides binding human PDZ domains. As in Figure 4, the domain is shown in surface representation, native and non-native motifs as ribbons, and key residues as sticks. The PDZ domain primarily binds C-termini of peptides. While the motif and particularly the key residues are fixed in the binding groove, the N-terminal context is much less restrained.

Yet the level of conservation of motif and context binding topologies varied considerably among families. Peptide ligands of Hormone_recep form α-helices which are structurally very similar, in the motif (1.9±2.1Å) as well as in the context (3.2±3.2Å). SH3 domains are special in this regard, because the different classes of peptides they bind have opposite directions with respect to the common poly-proline motif (Figure 3). The positions of the two prolines and the third key anchor residues ([RKY] or [RK], respectively) were relatively fixed in the structure, thereby also constraining the flexibility of the motif residues between them (RMSD 3.6±2.3Å). Class I ligands showed more flexibility than those in class II, though the small size of the data set does not allow us to establish whether this is a general trend. In both classes few constraints acted on the context, which often did not assume well-defined secondary structures and differed much more than the motifs (RMSD 6.7±4.4Å). The average motif RMSD for PDZ with 6.1±8.0Å was unusually high, possibly because of shifts in the ELM motif position. Nevertheless, visual inspection of the superimposed structures showed that the peptides all bound to the same pocket, and their positions did not differ greatly (Figure 4).

We also conducted a peptide exchange experiment in which we used the above structural domain superimpositions to assess the fit of each individual peptide onto every domain within the same family. We included all those interaction types with more than 10 distinct human domain-peptide pairs and, at least, one peptide of 10 or more residues, namely Cyclin_N, Hormone_recep, MATH, PDZ, SH2, SH3_1 class I and SH3_1 class II. We omitted EF hand because of its peculiar stoichiometry described above. We built the 6,738 non-native interaction pairs resulting from the combination of the above domains and interaction partners within each species, and computed both global binding energies as well as the contribution of individual positions to the interactions.

We saw that, 1536 out of the 6432 (24%) of the artificial (i.e. non-native) interactions tested have binding energies below the average of the native global energies for each type of peptide-mediated interactions. Moreover, we also observed that 1552 (24%) of them would bind better, with a lower energy, than the corresponding native pairs, indicating that interactions between these protein pairs would be possible, at least from a biophysical point of view. However, these numbers are likely to be overestimates as the vast majority peptide-mediated interactions that have been structurally analysed only contain truncated pieces of the full proteins involved in the interaction, which reduces the amount of context and thus its influence on the binding energy. Nevertheless, the most striking results arise from the differences observed between contacts involving residues in the motif vs the context. Here, we compared all the 6432 instances of artificial interaction pairs that showed a similar topological orientation of the contextual residues, as assessed by the motif RMSD. We found that, according to our energy calculations, one third (32.64%) of the linear motifs tested be compatible between different protein pairs, whereas this percentage drops to 16% when only the context contacts are considered. These two average values and, indeed, their corresponding distributions, are significantly different on a one-sided Fisher's exact test (p<2.2e^−16^), which supports the working hypothesis that context contacts are more specific than those found within binding motifs and thus play a key role in preventing potential cases of cross-reaction. Finally, it is worth noting that there is no correlation between unfavourable contextual interactions and large topological variations or with the length of the context.

**Figure 5:**
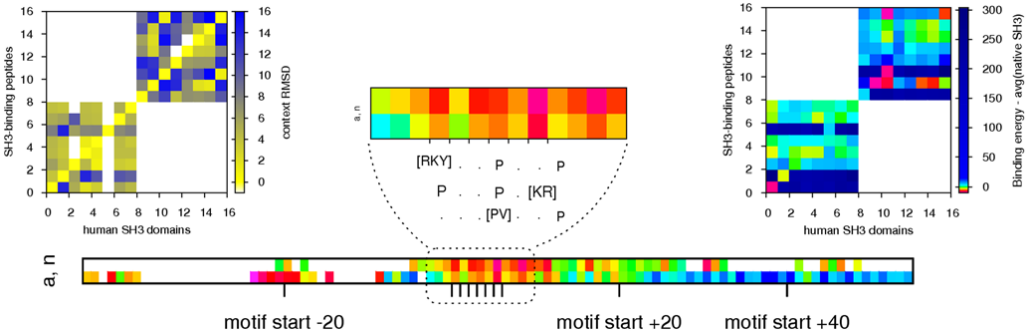
Peptide exchange results for human SH3 domains. Upper heat maps show the topological distortion (left) and the energy variations (right) of all artificial (i.e. non-native) interaction pairs constructed between human SH3 domains and their ligand proteins, with respect to the native topologies and the average native binding energy, respectively. SH3-binding peptides 1–9 correspond to class I and 10–16 to Class II. The lower figure shows the energy contribution of motif and context, with respect to the native binding energies, for each individual residue in the native (*n*) and artificial (*a*) interactions, determined by *in silico* alanine scanning. Detailed information is provided for the consensus motifs.

As for the interaction topologies, the mode of binding and the way to achieve specificity are often specific for each family. As mentioned above, there are two ways to orient peptides binding SH3 domains, and we analysed them separately. We found that 12% of the artificial interactions in human had a binding energy below the average of the native ones, and 15% bound better than the corresponding native interaction (Figure 5). The analysis by position showed that binding at the motif site was always good, in native but also in artificial cases. However, in the context several positions with sub-optimal contribution were observed, with increased frequency and strength for the non-native interactions. The results were similar for PDZ domains, with 7% non-native interactions with binding energies below the average of native PDZ-ligand-interactions, and 17% showing improved binding over the corresponding native interaction. The analysis of contribution by position again showed good binding for the motif in both native and constructed cases, and many unfavourable contributions in the context of non-native interactions (Figure S3). When we computed the energies for nuclear receptors binding either to NRBOX or CORNRBOX peptides (co-activators and co-repressors, respectively) we found the vast majority of them to be relatively low. 28% of the constructed interactions were below the average for native cases of this family, 23% bound better than the corresponding native cases, and the overview of all peptide exchange results showed that virtually all native and non-native interactions get good energy scores. The detailed interaction contribution profile highlighted the importance of the conserved leucines in the LxxLL pattern, but it also showed a high similarity between profiles for native and artificial interactions, which were more clearly separated for the two aforementioned domains. Also, contextual interactions were not as unfavourable as for the other domains. Together these observations suggest that the mechanism for specificity in interactions between nuclear receptors and cofactor peptides differs from that found for SH3 and PDZ, and that the former are more promiscuous.

Finally, we compared the results obtained in the peptide exchange experiment with pairwise sequence identities between the native and non-native binding partners to see whether there was a direct relationship between sequence similarity at peptide and domain level and interaction exchangeability (Figure S4). Considering the average binding energy for each type of native interaction as the threshold for feasibility, we observed that highly similar instances (at least 80% sequence identity and peptide similarity) allowed for mutual exchange of binding partners in only 15% of the cases, ranging from 0% to 33% in the different families. Furthermore, we also observed that 62 cases (5%) with sequence identities below 30% allowed for uni- or bi-lateral exchange, thereby making it extremely difficult to predict potential cross-reactions from sequence alignments alone.

## Discussion

To the best of our knowledge, this is the first time that high-resolution three-dimensional structures have been systematically employed to study transient peptide-mediated protein interactions. The exhaustive compilation and analysis of all the instances in the PDB have partially revealed the molecular mechanisms used by evolution to achieve the dynamic nature and specificity required in this type of interaction. More specifically, our study has quantified the energetic contribution of the interaction consensus motif and context residues, respectively, to binding. Finally, we have also studied and quantified, from topological and energetic perspectives, the relationship between context and interaction specificity.

The first conclusion drawn, in light of our findings, is that the definition of several classical interaction motifs should be revised, and some new ones identified in this study should be included in the respective resources.

However, the most striking results come from the quantification of the binding energy contributions and their implications. Our results convincingly show that the contribution of motif residues to global binding energy is paramount to ensure interaction, while the contextual contacts are most likely used to achieve high specificity. These observations suggest that contacting residues involved in peptide-mediated interactions have been selected through evolution on two different bases: those in the motif to ensure binding with high affinities, and those in the context to maximise specificity against other potential binders and prevent cross-reaction with homologous proteins. The “negative selection” evolutionary model for context residues was previously proposed by Lim and co-workers based on SH3 domains in yeast and a 10-residue peptide matching the SH3 class I-motif [14]. Our results support the general validity of this model for peptide-mediated interactions. It has also been proposed that some domains binding linear motifs rely on sub-optimal contacts to achieve the affinity and kinetic constants (i.e. K_on/off_) necessary to perform their signalling functions [9]. Our findings also support the concept of sub-optimal contacts in domain-peptide interaction interfaces and, furthermore, show that the motif often forms stronger contacts, whereas the sub-optimal contacts in the context are crucial for specificity. We have observed that some residues in native interfaces have a disturbing effect on the interaction, and that their replacement by other residues increases the binding energy, leading to lower dissociation constants (K_d_s). However, these sub-optimal contacts have a much stronger effect in non-native interactions, where they completely disrupt the potential interfaces, preventing thus cases of potential cross-reaction.

Concerning the interaction topology, we show that the position of the motif is conserved in the structure, while more flexibility is allowed for the placement of the context. It should, however, be taken into consideration that many structures in our dataset were determined using only a fraction of the actual binding partner, allowing for more flexibility in the protein's termini, which would be restrained in the full protein. This effect is especially pronounced in structures solved by NMR. Furthermore, it should be considered that interactions between full-length proteins are likely to show a stronger effect of the context, because the interaction surfaces will increase. The FHA-motif interactions discussed above give an example of how a larger context can influence the interaction.

In some interaction types, such as those involving SH2 domains, the binding consensus motif is too short and degenerated to draw any significant conclusion. Besides, these interactions often depend on phosphorylation events and thus specificity is very likely to come from other biological context [16]. In the case of MATH, the limited sequence diversity probably restricts the expressiveness of our results. MATH:TRAF interactions occur in trimers *in vivo*, and it has been suggested that, while affinity and specificity for single interactions are not high, the trimerization amplifies both aspects and can thus lead to highly specific interactions [26].

Comparing the contribution of context to binding and its length as determined in the structure, we observed an increase in the contribution of longer peptides, which was not related to the size of the source protein from which the peptide was taken. We therefore assume that the true contextual contribution may well be higher than what we observed here, due to a bias towards short peptides in current structures of transient interactions.

Our peptide-exchange experiment showed that some non-native peptide-mediated interactions are energetically possible, but may have weaker binding energies than native protein pairs. It is worth remembering that, in this experiment, we tested only possible cases of potential cross-reaction between protein pairs of known 3D structure within species, regardless of other key aspects in biological regulation such as sub-cellular localization or expression times. And yet, we identified several cases where cross-reactions within the same sub-cellular compartments seem to be possible. These instances could represent potential backup circuits to increase the robustness of protein-protein interaction networks, since they could compensate individual protein failures [27], [28].

The occurrence of (seemingly) energetically feasible non-native pairings was observed across all families studied here, but we found that non-native pairings with low energies are quite common between nuclear receptors (NR) and cofactor peptides. Nuclear receptors have 4 interaction interfaces: a ligand-binding pocket that holds lipophilic compounds (e.g., hormones like oestrogen), the cofactor binding groove, a dimerization interface, and a DNA-binding site binding the HRE (hormone response element) for its activity as a transcription factor. Our studies here addressed only the cofactor peptide. While specificity for the hormone ligand is usually high [29], at least some co-activators have been found to bind promiscuously. In general, binding of the hormone ligand induces a specific position of the C-terminal helix H12, which in turn allows the co-activator peptide to bind; however overexpression of the co-activator may also lead to an active nuclear receptor, despite lack of the ligand [30]. Co-repressors prefer a different position of the C-terminal helix and may interact with apo-NRs, or those binding an antagonist ligand. A recent study showed that it may not be as uncommon for peptides to bind to apo-NRs, but also reported increased affinity in the presence of ligand: the authors tested the effects of 11 ligands and 52 peptides on a selected receptor, and proposed that the effect on gene expression is a result of the combination of the ligand and the co-activator/-repressor peptide [31]. Hence there are several factors that determine whether, and how strong, a cofactor can bind, the motif and flanking sequences are just some of them. The tertiary structures of the NR were not modified in the peptide exchange, thus H12 was fixed, and 70 out of 72 of our non-redundant native structures bound co-activators. This may be the main reason why we observed a good binding for most cases, and a high percentage of expected cross-talk. Binding energies for the 2 co-repressors in co-activator-binding structures were worse. In addition, tissue-specific expression has also been observed for cofactors [30], which is beyond the criteria the peptide exchange considers, and may be another cause of potential cross-reactivity that does not have an *in vivo*-effect and thus binding patterns do not evolve against. All together, we conclude that other factors such as the hormone ligand and the tertiary structure of the NR are crucial players in cofactor binding, and that the contextual interactions are not as important as for other families.

The identification of potential non-native interactions and putative backup circuits is paramount to understand how cell networks work as a whole, in what it is known as systems biology. Given that we did not observe a clear correlation between sequence identity and binding for any of the cases studied, we conclude that sequence information alone is insufficient to make predictions on domain-peptide interactions. Thus knowledge or modelling of the interacting structures is required in order to successfully predict whether a given domain-peptide-pair will bind or not; similar approaches for the prediction of domain-domain interactions have proven successful [32], [33]. The observation that the interface position and binding sites for key residues are structurally conserved will simplify the development of such a predictive tool, although Nature has other means to prevent undesired cross-talk between cellular processes [34].

Knowledge of the atomic details as to how transient protein interactions occur and the ability to predict peptide-mediated protein interactions are crucial for understanding and modelling regulatory processes, for the design of new cellular circuits in synthetic biology and the discovery of drugs that target such interactions [35]. We believe that the mechanisms of binding and specificity reported here will make a considerable contribution to these areas.

## Materials and Methods

### Identification of peptide-mediated protein interactions of known 3D structure

To detect all cases of peptide-mediated protein interactions of known 3D structure, we first parsed the PDB (02/2007) and identified all those entries containing two or more interacting proteins. We extracted all the information regarding the different 66 types of ligand involved in peptide-mediated interactions from the Eukaryotic Linear Motifs (ELM) database (03/2007) and assigned Pfam families [36] to all the globular domains involved in the interactions via literature curation. We then used BLAST (Evalue≤0.01) to assign Pfam families to all interactions of known 3D structure. Whenever we identified a protein chain containing an ELM-binding domain, we searched all contacting chains for occurrences of the linear consensus motif. When we found a motif match in close vicinity of the globular domain (≤10 Å) we considered it a potential domain-peptide interaction. Finally, we went manually through the 2200 potential hits, comparing the interacting structures to those described in the literature, and removing false positives where the interaction was not mediated by the consensus peptide. Because of the visual inspection we are confident that the interactions reported here are biologically relevant and not artefacts that might arise e.g. from crystal packing.

To avoid composition biases, we created a non-redundant set of interactions by clustering those pairs sharing a 100% sequence identity on both the domain and the peptide.

### Computation of binding contribution

To quantify the contribution of motif and context to the interaction, we used the FoldX package [37], [38] to conduct *in silico* alanine scanning experiments. FoldX is an empirical force field which combines physical descriptions of interactions between residues with experimentally determined results. It takes into account solvent exposure, hydrogen bonds, electrostatics, van der Waals energies and clashes, water bridges, and backbone and side-chain entropy. The FoldX force field was initially trained on a set of over 300 mutants and tested on a set of over 600 mutants as well as on 82 protein-protein interaction interfaces, with a reported correlation between calculated and experimentally determined folding and binding energies of 0.8. SH3-peptide interactions, which we also studied in this work, were among the set of protein-protein interfaces. Furthermore the force field was shown to account for both stabilizing and destabilizing mutations with a high correlation (0.89) with experimental results [37]. More recent developments of the program have further improved the mutation prediction accuracy for single proteins as well as interactions. While deviations of the predicted values from experimental individual single mutation results have been observed, FoldX performs well in identifying trends of effects of mutations and has been successfully used in several studies, in combination with homology modelling, to identify potential binding partners of a given protein [33], [39], [40]. We first computed the binding energy of the native interaction interface. We then truncated either the motif or the context residues to alanine and re-estimated the binding energy for the new interface, in order to estimate the relative binding contribution of each section by comparison with the energy of the native interaction. We also analysed the contribution to the global energy of each residue independently (“complex_alascan” [38]) via alanine scanning, which tests for stabilizing and destabilizing effects as well as for their magnitude compared with other residues. To ensure equal conditions for all structures, we applied relaxation to the interface before each energy computation, to optimise positioning of the side chains and remove any distorting effects that mutations may have introduced, using the strong force field of FoldX (option VdWDesign = 2, which assigns strong repulsive energies to Van der Waals clashes). Explicit relaxation of a given structure is possible only in a pre-release version of the force field we obtained from the developers, though it is included in the complex_alascan analysis [38]. Relaxation eliminates possible artificially high energy values caused by problems with side chain positioning in the original or modified structure; the backbone conformation is not changed. Arginine side chains were excluded from relaxations upon recommendation of the FoldX developers, since they may lead to non-optimal solutions (François Stricher, personal communication). It is worth noting that most of our analyses are entirely based on energy differences rather than on absolute energy values, so they are mainly qualitative and thus less affected by inaccuracies of empirical force fields.

### Position specific sequence conservation vs binding contribution

To test for a potential correlation between position specific sequence conservation and individual contribution to the binding energy, we computed the entropy of each residue in a multiple sequence alignment [41] and compared the values to the alanine scanning results obtained in our analysis. We calculated three entropy values for each position based on the Pfam [36] “seed” and “full” alignments, and on a profile-based multiple alignment manually derived from the sequences present in our non-redundant set of peptide-mediated interactions of known structure.

To analyse whether the contribution of motif- and context-binding residues to the binding energy in the globular domain differs, we computed the “context binding fraction” for each domain position, that is, the ratio of context contacts vs. all interchain (i.e. motif plus context) contacts observed for this residue. To study differences in binding contribution in the peptide, we split the ELM pattern into its positions and grouped them on the basis of stringency: “fixed”, “restricted”, and “arbitrary”, if only one, a small set, or any amino acid is allowed in this position of the motif, respectively. We then compared the alanine scanning results for motif positions as classified above.

### Peptide exchange

We performed a peptide exchange experiment for each non-redundant set of interaction pairs with 10 or more representative 3D structures, and where at least one peptide was sufficiently long for studies of contextual effects (≥10 residues). For each pair of interactions (d1:p1, d2:p2), we structurally superimposed the globular domains (d1, d2) using STAMP [42] and applied the same transformation to the ligands so that they were positioned in the appropriate binding groove of the non-native domain (d1:p2, d2:p1). Like for the native interactions, we then relaxed the new interaction pairs using the strong force field option to remove clashes and compute the binding energies with the less strict force field (VdWDesign = 0). We also used these transformations to calculate the Cα RMSD between the motif and context residues of the native and chimera protein pairs. Subsequently, we estimated the putative binding energies for all non-native protein interaction pairs, and the contribution of motif and context as well as individual positions through *in silico* alanine scanning experiments, as described above.

We predicted potential non-native interactions when the binding energy of the constructed pair was either lower than that of the native peptide, or below the average of all native interactions of a particular interaction type (domain-peptide pair). We defined cross-talk as unidirectional when only one of the constructed interactions (d1:p2 or d2:p1) had a sufficiently low binding energy, or bidirectional, when both constructed interactions bound strongly enough. Pairwise domain sequence identity and peptide similarity were determined via Needleman-Wunsch global alignment [43]. Global binding energy results are sorted by sequence identity using the same algorithm and clustered by the program Neighbor from the PHYLIP package [44].

## Supporting Information

Table S1 List of all domain-motif interactions of 3d structure
(0.10 MB TXT)

Figure S1 Percentage of fixed, restricted, and arbitrary motif positions observed in our structural data and their corresponding binding contribution, determined via glycine scanning. Contribution between 0 and 2 is frequently observed for all three types, but fixed positions show a contribution between 2 and 4 much more often than arbitrary residues.
(0.02 MB EPS)

Figure S2 Distribution of the RMSDs observed for motif (solid lines) and context (dashed lines) among families studied in the peptide exchange. The inset shows a zoom to the RMSD range [0,10], where the majority of both motif and context differences are found. Motif RMSDs above 5 Å are rare, and only domains binding large ligands (PDZ, efhand) show a few cases with contextual RMSDs above 20 Å.
(0.04 MB EPS)

Figure S3 Contribution of each relative position in the motif in binding of peptides to PDZ domains, native (n) and artificial (a) interactions as constructed in the peptide exchange (see Materials and Methods). Red through yellow indicated good binding, green is neutral, cyan and blue indicate unfavorable interactions. The motif with its consensus patterns and a few surrounding residues are specifically highlighted. One of the three patterns allows internal peptides, while the other two require the motif to be C-terminal, indicated by a $ at the end.
(5.08 MB TIF)

Figure S4 Exchangeability across all families studied in the peptide exchange. If both non-native protein pairs have a binding energy below the average of the native cases of the corresponding family, we predict bidirectional exchange (magenta squares). If only one of the non-native combinations is below this threshold, we predict unilateral exchange (blue circles). If none of the artifical pairs has a binding energy below the average of the native cases, we predict no cross-talk (cyan diamonds).
(4.73 MB TIF)
