# Supplementary figures and images for: Contextual Specificity in Peptide-Mediated Protein Interactions
Source: PLoS One. 2008 Jul 2;3(7):e2524. doi: 10.1371/journal.pone.0002524 (PMC2438476; doi:10.1371/journal.pone.0002524)

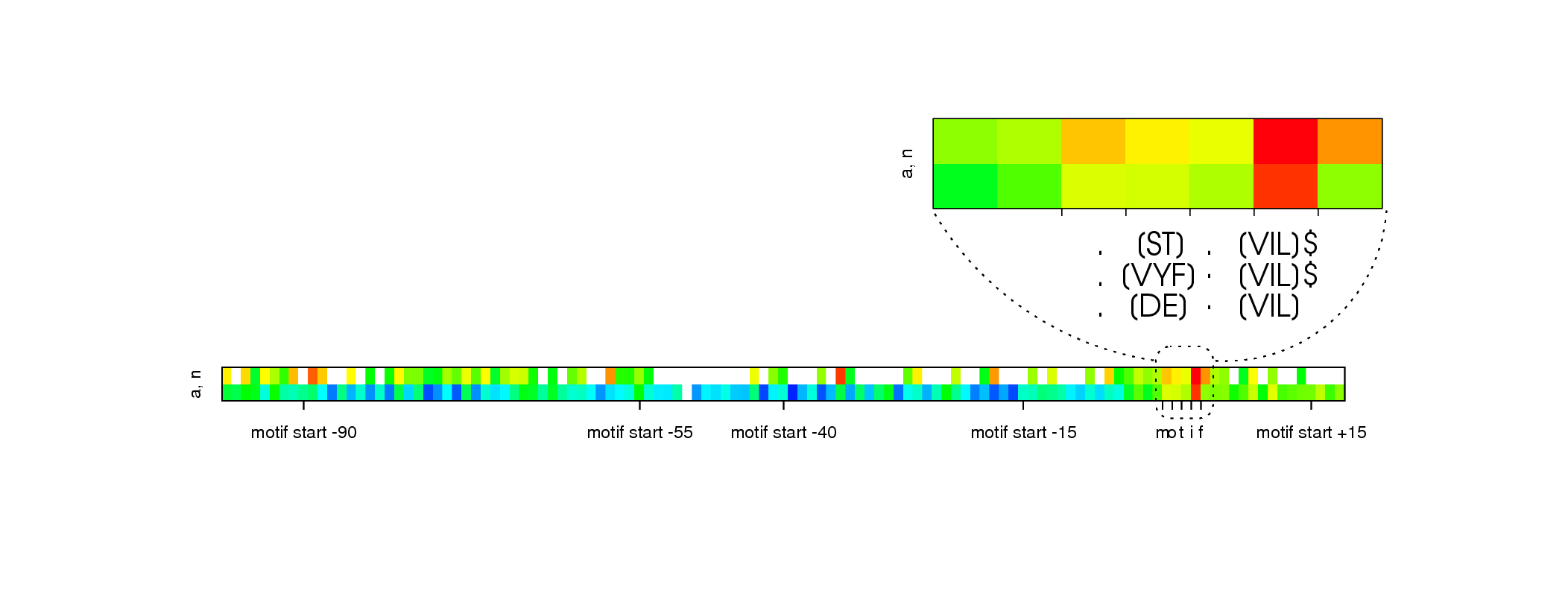

Supplement: Figure S3 — Contribution of each relative position in the motif in binding of peptides to PDZ domains, native (n) and artificial (a) interactions as constructed in the peptide exchange (see Materials and Methods). Red through yellow indicated good binding, green is neutral, cyan and blue indicate unfavorable interactions. The motif with its consensus patterns and a few surrounding residues are specifically highlighted. One of the three patterns allows internal peptides, while the other two require the motif to be C-terminal, indicated by a $ at the end. (5.08 MB TIF) [file pone.0002524.s004.tif]

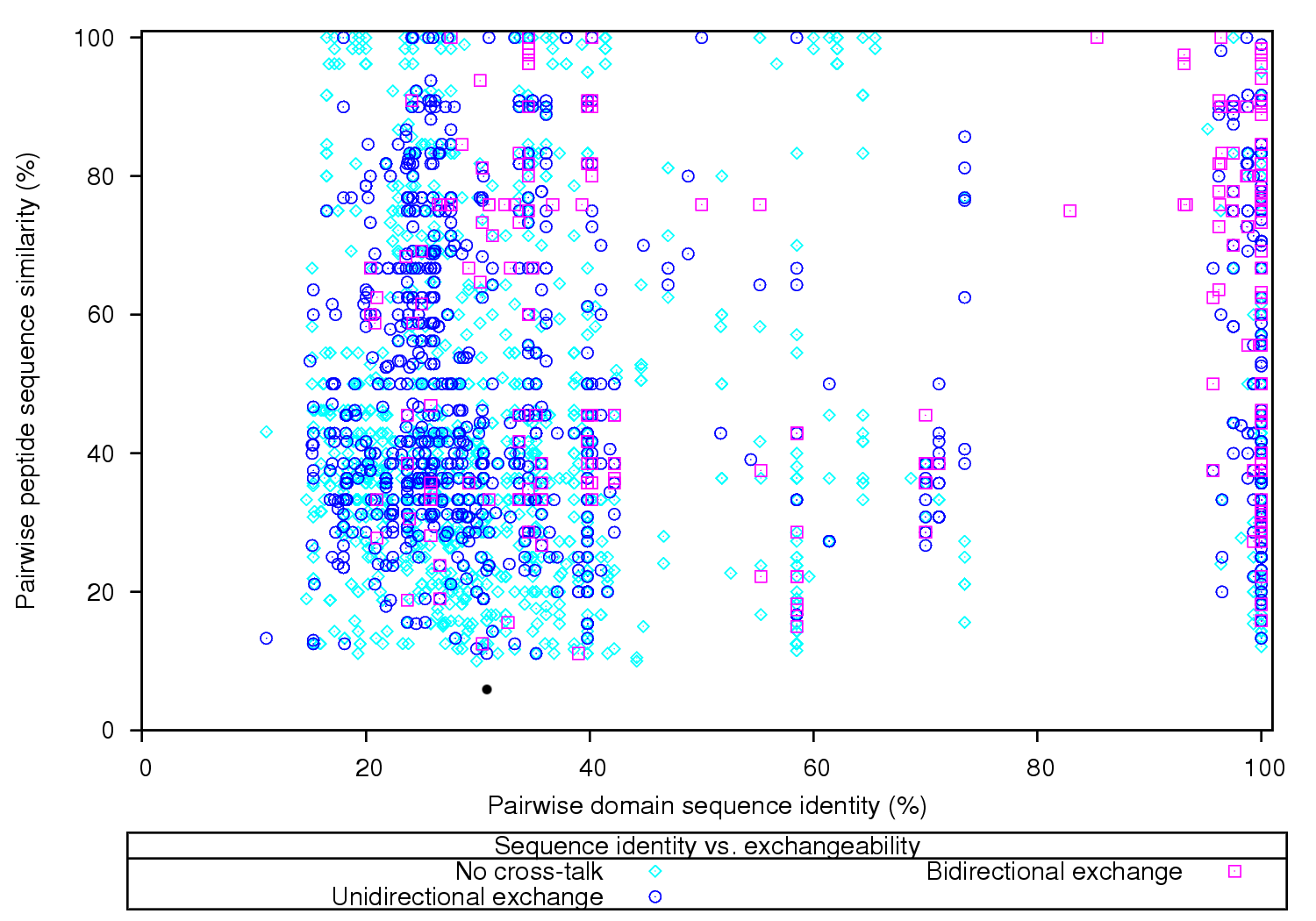

Supplement: Figure S4 — Exchangeability across all families studied in the peptide exchange. If both non-native protein pairs have a binding energy below the average of the native cases of the corresponding family, we predict bidirectional exchange (magenta squares). If only one of the non-native combinations is below this threshold, we predict unilateral exchange (blue circles). If none of the artifical pairs has a binding energy below the average of the native cases, we predict no cross-talk (cyan diamonds). (4.73 MB TIF) [file pone.0002524.s005.tif]
